# The Power of Belief: Investigating the Placebo Effect in Post-Exercise Recovery Strategies for Football Players

**DOI:** 10.3390/healthcare14010004

**Published:** 2025-12-19

**Authors:** Marco Pernigoni, Andrius Osvaldo Alfieri, Audinga Kniubaitė, Sigitas Kamandulis, Daniele Conte, Inga Lukonaitienė

**Affiliations:** 1 Department of Coaching Science, Lithuanian Sports University, 44221 Kaunas, Lithuania; andrius.alfieri@stud.lsu.lt (A.O.A.); audinga.kniubaite@lsu.lt (A.K.); daniele.conte@uniroma4.it (D.C.); inga.lukonaitiene@lsu.lt (I.L.); 2 Institute of Sport Science and Innovations, Lithuanian Sports University, 44221 Kaunas, Lithuania; sigitas.kamandulis@lsu.lt; 3 Department of Movement, Human and Health Sciences, University of Rome “Foro Italico”, 00135 Rome, Italy

**Keywords:** athletic performance, fatigue, treatment expectations, sport psychology, team sports

## Abstract

**Objectives**: The objective is assessing whether the placebo effect can influence the time course of recovery following a football match. **Methods**: Using a randomized crossover design, eighteen youth male players (age: 15.3 ± 0.5 years, stature: 178.7 ± 6.4 cm, body mass: 65.3 ± 7.6 kg, playing experience: 8.6 ± 1.5 years) completed two friendly matches, followed by placebo (PLA; sham vagus nerve stimulation) or passive rest (CON). To assess the impact of PLA, countermovement jump height (CMJ), 10 and 20 m sprint times, heart rate variability (Ln-rMSSD), static and dynamic muscle soreness, and perceived fatigue were measured at pre-match, post-match, post-recovery, and 24 h post-match. **Results**: Our findings indicate that match play induced substantial fatigue, with significant deteriorations [*p* ≤ 0.002, *small-to-large* effect sizes (ES)] in CMJ, 10 and 20 m sprint performance, Ln-rMSSD, muscle soreness, and perceived fatigue at post-match and post-recovery compared to pre-match (except Ln-rMSSD between pre-match and post-recovery: *p* = 0.151, *small* ES). Although no significant between-intervention differences were found for any variable at any time point (*p* > 0.05), effect size analysis showed *moderately* lower perceived fatigue (*r* = 0.40) and dynamic soreness (*r* = 0.32) in PLA compared to CON at post-recovery. **Conclusions**: These findings suggest that while placebo stimulation did not affect performance or heart rate variability, it may support perceptual recovery. This holds relevance for both research and practice, as including placebo conditions can help isolate psychological effects from true treatment responses, while promoting positive expectations may enhance the perceived effectiveness of recovery strategies.

## 1. Introduction

The demanding nature of sport activity can generate substantial fatigue in athletes, due to intense physical demands and the high frequency of modern training and competition [1]. As a result, the use of recovery strategies has attracted growing interest from practitioners aiming to reduce the negative effects of accumulated fatigue [2].

In the modern sport environment, a considerable number of recovery tools are available to practitioners [3]. Specifically, strategies like cold water immersion, massage, compression garments, and nutritional interventions may enhance post-exercise recovery through physiological mechanisms like reduced inflammation and muscle damage, improved muscle blood flow, and metabolite clearance [2]. However, it has been suggested that the real-world effectiveness of these strategies may also be driven—at least in part—by placebo effects rather than solely physiological mechanisms [4,5,6,7].

Overall, the role of the placebo effect in sport has been widely investigated, with evidence indicating that it can enhance perceived well-being and directly benefit performance [8,9]. These effects have been attributed to positive expectations, which trigger neurobiological (e.g., increased dopamine, opioids, and endocannabinoid activity) and psychological responses that modulate motivation, pain, and fatigue [10], ultimately enhancing performance [8]. However, the role of the placebo effect in recovery strategies has received limited attention. Specifically, although countless studies have used placebos (e.g., capsules, beverages, sham electrostimulation) as control interventions, few have quantified the “true” magnitude of the placebo effect, defined as the difference between a placebo intervention and changes observed in a control group receiving no treatment [9] (i.e., passive rest). Moreover, the available research in this area has produced conflicting findings, with one study reporting no differences between placebo and passive rest [11], another showing placebo-related improvements in performance and perceptual markers [5], and a third not providing direct statistical comparisons [12].

Taken together, these findings suggest that the placebo effect may contribute to the effectiveness of recovery strategies, but also highlight the uncertainty that still surrounds this topic. Hence, understanding how the placebo effect influences post-exercise recovery has important implications for both research (to isolate and quantify placebo effects) and practice (to provide further insights into the factors affecting recovery). These considerations are particularly relevant in team sports, where athletes are often exposed to high-intensity exercise, coupled with congested competition schedules [1]. These conditions not only increase the need for effective recovery strategies, but also provide a suitable setting for examining the placebo effect in ecologically valid, real-world contexts.

Therefore, this study aimed to investigate the effect of a post-exercise placebo intervention (presented as non-invasive vagus nerve stimulation) on performance [countermovement jump height (CMJ), 10 and 20 m sprint times], physiological [heart rate variability (HRV)], and perceptual (muscle soreness and perceived fatigue) measures of fatigue following a friendly match in youth football players. It was hypothesized that—compared to passive rest—the placebo intervention would produce improvements in performance and perceptual recovery, while HRV analysis was treated as exploratory.

## 2. Materials and Methods

### 2.1. Design

Prior to the beginning of the study, participants were familiarized with all procedures and were told to maintain a stable diet (i.e., without excessive variations in day-to-day food intake) and follow their regular sleep routines throughout the study period.

**Figure 1:**
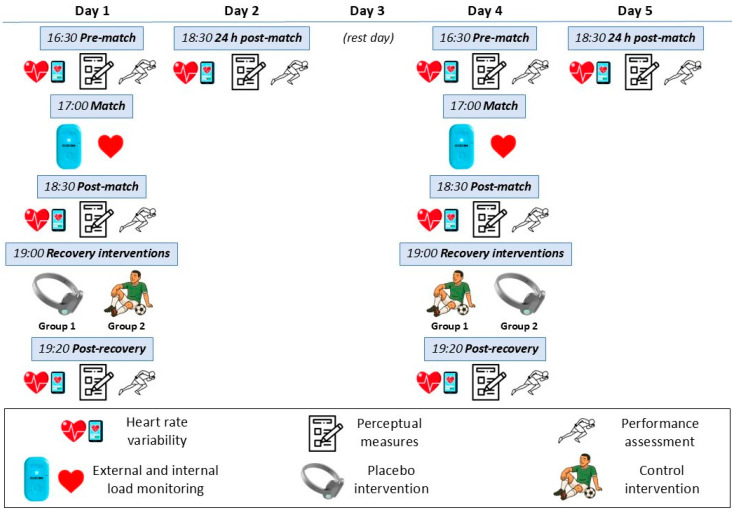
Study timeline and procedures.

A randomized crossover design was used, with all participants completing both conditions [placebo intervention (PLA) and passive rest (CON)], and the order of these conditions was randomly assigned for each player (Figure 1). Specifically, participant assignment was performed using a freely available online tool (https://www.randomlists.com/team-generator, accessed on 7 March 2025). All players took part in two friendly football matches (i.e., one for each condition; both played on an outdoor pitch and separated by a 72-h washout period), followed by either PLA or CON. For each match, measures of performance (CMJ, 10 and 20 m sprint times), physiological (HRV), and perceptual fatigue (muscle soreness and perceived fatigue) were assessed at pre-match, post-match, post-recovery, and 24 h post-match. All these measurements were conducted indoors, within a gym complex adjacent to the football pitch. At each time point, assessment was performed following a standardized sequence as follows: physiological measures were collected first, followed by perceptual measures and—finally—performance assessment (to avoid any influence of performance testing on physiological or perceptual outcomes). At pre-match, assessments began ~30 min before match-start, with post-match and post-recovery assessment performed immediately after match-end, and immediately after completion of PLA/CON, respectively. During both matches, external and internal loads were also monitored.

To minimize bias stemming from potential differences in fatigue status at baseline (i.e., pre-match) and 24 h post-match, players were instructed to refrain from physical activity for 48 h before each match and for 24 h afterward, until all measurements (i.e., performance, physiological, and perceptual assessment) were completed.

### 2.2. Participants

**Figure 2:**
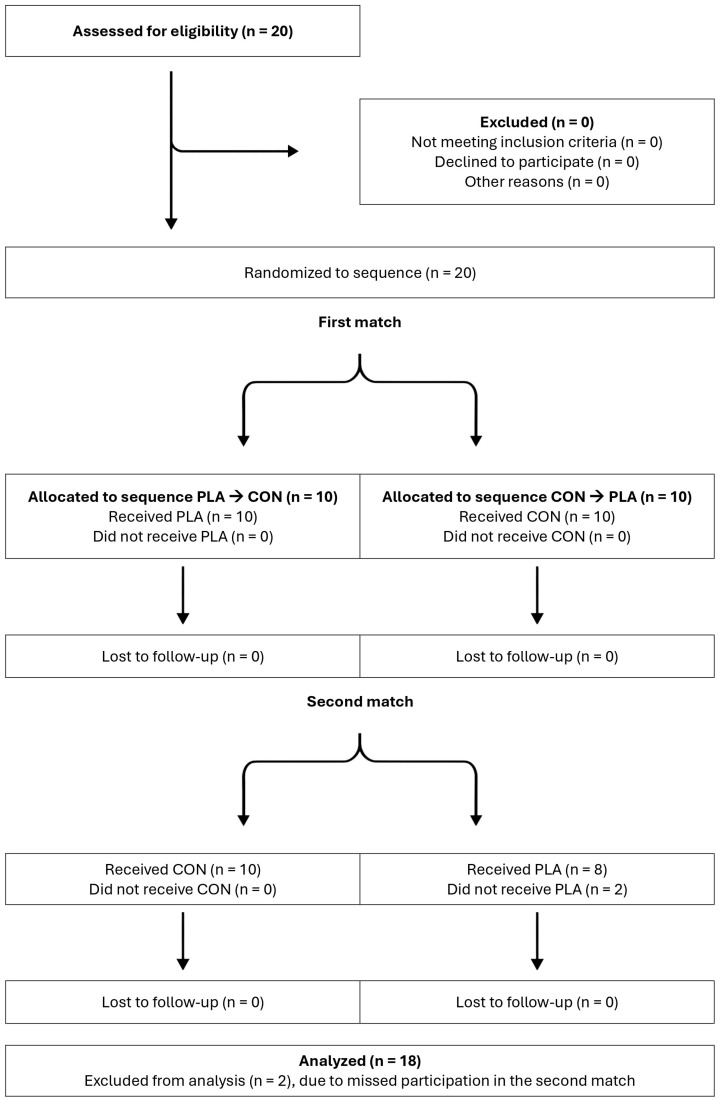
Participant flow diagram at different stages of the study (template adapted from Dwan et al. [13]). Abbreviations: PLA, placebo intervention; CON, control intervention.

Figure 2 reports the participant flow diagram for the present study.

Twenty youth male football players were initially recruited for this study. Inclusion criteria comprised the following: (1) participants had to be part of their team (i.e., no team transfers or dropouts) for the entire duration of the study and complete the two football matches; (2) participants had to be injury-free across the experimental period; (3) participants had to be outfield players (i.e., not goalkeepers). During the study, two players were excluded due to missed participation in the second match. Therefore, 18 players (age: 15.3 ± 0.5 years, stature: 178.7 ± 6.4 cm, body mass: 65.3 ± 7.6 kg, football playing experience: 8.6 ± 1.5 years) were retained for analysis. A priori power analysis (G*Power version 3.1.9.7, University of Düsseldorf, Germany) indicated that this sample size was adequate (minimum *n* = 12) using α = 0.05, power (1−β) = 0.80, and effect size *f* = 0.29, based on a similarly designed research [5]. During the study period, the players’ typical weekly schedule included four 2-h training sessions and one competitive match. After the explanation of the experimental design, written consent was obtained from the parents/guardians of all participants, who were informed of their right to refuse participation or withdraw from the research at any time. The study was approved by the Lithuanian Sports University Ethics Committee [NR. BNL-TRS (B)-2025-773, approval date 6 March 2025; study start 14 March 2025] and designed according to the Declaration of Helsinki.

### 2.3. Procedures

#### 2.3.1. Friendly Football Matches

The two friendly matches (11-vs-11) consisted of two 45-min halves (without additional time), separated by a 5-min halftime. Both matches were refereed by a member of the coaching staff and played on the same 105 × 68 m artificial turf pitch used during the team’s league games. Ambient temperature and humidity were 1 °C and 100% for the first match, and −1 °C and 69% for the second, respectively, with both matches played in dry weather (i.e., no rain or snow). The two players who dropped out after the first match (see Section 2.2) were replaced by players of similar age and competitive level (not included in the analysis) in the second match. All participants played the entire duration of each match.

#### 2.3.2. Post-Match Placebo Intervention

Approximately 30 min after the end of each match, players received either the PLA or CON intervention in one of two separate rooms located within the aforementioned gym complex.

Before the study, participants were informed that the research aimed to assess the effect of a non-invasive vagus nerve stimulation device (Pulsetto, Klaipėda, Lithuania) on post-exercise recovery, and were provided with a description of its benefits. However, during the PLA intervention, only sham stimulation was administered. Specifically, participants sat for 20 min while wearing the device, which was turned on (to mimic normal operation) but not activated (i.e., no stimulation program was selected, resulting in no current being delivered). This aspect was disclosed to participants only after the conclusion of the study.

Conversely, during the CON intervention, players remained seated on chairs in a quiet indoor room for 20 min, without any device applied and without engaging in any activity.

#### 2.3.3. Performance Assessment

Performance measurements at all time points (except for post-match) were conducted after a ∼10 min standardized warm-up (Supplementary Materials, Table S1), to ensure that potential changes between time points were due to fatigue-related factors and not drops in muscle temperature and/or activation.

The CMJ test without arm swing [14] was used to assess jumping performance through the Optojump system (Microgate, Bolzano, Italy). Briefly, players started in the erect standing position and were told to jump “as high and as fast as possible” using a self-selected countermovement depth.

Additionally, 10 and 20 m maximal sprint times were recorded during a 20 m sprint test with a 10 m split. Specifically, three timing gates (Witty, Microgate, Bolzano, Italy) were positioned at 0, 10, and 20 m from the start, with players beginning their sprints 50 cm behind the first gate (to prevent accidental triggering of timing in the start position).

At each time point, participants performed three CMJ trials (interspersed by ∼1 min of passive rest) and two sprint trials (∼2 min passive rest), with the best result used for analysis. The reliability of the employed CMJ [intraclass correlation coefficient (ICC) = 0.99; standard error of the measurement (SEM) = 0.79 cm; coefficient of variation (CV) = 3.70%] [15] and sprint (ICC = 0.92–0.95) [16] testing procedures has been previously established.

Of the 144 scheduled measurements for each test (i.e., jumping and sprinting), 2 were missing due to participants’ unavailability (i.e., both at 24 h post-match).

#### 2.3.4. Physiological Assessment

Bluetooth heart rate (HR) monitors (HRM-Dual, Garmin Ltd., Olathe, KS, USA) were used to record HRV in supine position. Participants measured HRV for 90 s via the Elite HRV application (Ashville, NC, USA), whose validity (*r* = 0.85 compared with gold-standard electrocardiogram measurement) and reliability [ICC = 0.94; SEM = 4.16%; minimal detectable change (MDC) = 11.52%] have been established in previous research [17]. Specifically, the log-transformed squared root of the mean sum of the squared differences between R-R intervals (Ln-rMSSD) was calculated to investigate parasympathetic autonomic activity.

The application includes an in-built function that automatically detects and corrects artifacts and provides a signal quality rating (“good”, “okay”, or “poor”). To ensure accuracy, only recordings rated as “good” were considered for analysis. Accordingly, 3 measurements (out of the total 144 scheduled) were excluded due to “okay” or “poor” signal quality, with 2 further measurements excluded due to participants’ unavailability (as mentioned earlier).

#### 2.3.5. Perceptual Assessment

As previously described [18,19], Borg’s CR10 scale of perceived muscle pain for lower limbs between 0 (“nothing at all”) and 11 (“maximum pain”) was used to assess muscle soreness in a static condition (i.e., standing without moving). Moreover, muscle soreness in the quadriceps muscles was assessed in a dynamic condition (i.e., while performing a deep squat), using a previously employed scale from 0 (“none”) to 10 points (“intolerably intense”) [20]. Finally, the overall perception of fatigue was measured using the validated Rating-of-Fatigue scale [21], ranging between 0 (“not fatigued at all”) and 10 (“total fatigue and exhaustion—nothing left”).

#### 2.3.6. External and Internal Load Monitoring

External load was monitored using WIMU PRO devices (Realtrack Systems SL, Almería, Spain) as previously described [22]. Briefly, match Player Load [PL, arbitrary units (AU)] was calculated from changes in tri-axial accelerations during exercise, and PL/min (AU/min) was obtained by dividing PL by match duration. Additionally, the total distance (m) covered by each player was recorded. Regarding internal load, the highest HR value recorded during any of the two matches (HR_peak_) was identified for each player, and exercise intensity was calculated as the average percentage of HR_peak_ throughout each match (%HR_peak_). Finally, Rating of Perceived Exertion (RPE) scores were collected approximately 10 min post-match through the modified CR10 scale [23], and then multiplied by match duration (min) to determine session-RPE load (s-RPE, AU).

### 2.4. Statistical Analysis

Descriptive statistics [mean ± standard deviation (continuous data) or median ± interquartile range (ordinal data)] were calculated for each variable.

Separate linear mixed models—which correctly deal with missing values and repeated measures—were used for each continuous dependent variable (CMJ, 10 and 20 m sprint times, Ln-rMSSD) to calculate the effect of time, intervention, and time*intervention interaction. In these models, time and intervention represented the fixed effects, while player represented the random effect. In case of statistically significant differences, Bonferroni post hoc analyses were run. All variables showed normally distributed residuals (Shapiro–Wilk test *p* > 0.05).

To assess the effect of time for ordinal data (muscle soreness and perceived fatigue), separate Friedman tests (i.e., one for each intervention) were used. When the effect of time was statistically significant, post hoc Wilcoxon tests with Bonferroni correction were used. The same test and correction were also used to compare ordinal data between interventions at corresponding time points. As previously noted, two participants were unavailable at 24 h post-match. Therefore, perceptual data were reported for 16 players (rather than the 18 included in the continuous-variable analyses), since appropriate nonparametric methods for handling missing values in ordinal data were not available (as opposed to continuous variables).

After assessing the normal distribution assumption via the Shapiro–Wilk test (*p* > 0.05), differences in PL, PL/min, total distance, and %HR_peak_ between PLA and CON were assessed using paired-samples t-tests, while non-normally distributed s-RPE data (*p* < 0.05) were compared through the Wilcoxon test. Due to the limited number of available WIMU PRO devices, PL, distance, and HR data were initially collected from 15 players. However, due to dropouts (*n* = 2), the final analysis was performed on 13 players.

The magnitude of differences for pairwise comparisons was assessed using Cohen’s *d* [with 95% confidence intervals (CI)] for parametric analyses and was interpreted as *trivial* (<0.20), *small* (0.20–0.59), *moderate* (0.60–1.19), *large* (1.20–1.99), and *very large* (≥2.0) [24]. For non-parametric analyses, ES was calculated using the *r* value (Wilcoxon z value/√N) and interpreted as *trivial* (<0.10), *small* (0.10–0.29), *moderate* (0.30–0.49), and *large* (≥0.50) [25]. Statistical significance was set at *p* < 0.05 and all analyses were carried out using the Jamovi software package for Windows^®^ (version 2.3.28, Sydney, Australia).

## 3. Results

Table 1 reports external and internal load data for each match. Non-significant (*p* > 0.05), *trivial* differences were found between interventions for all variables.

**Figure 3:**
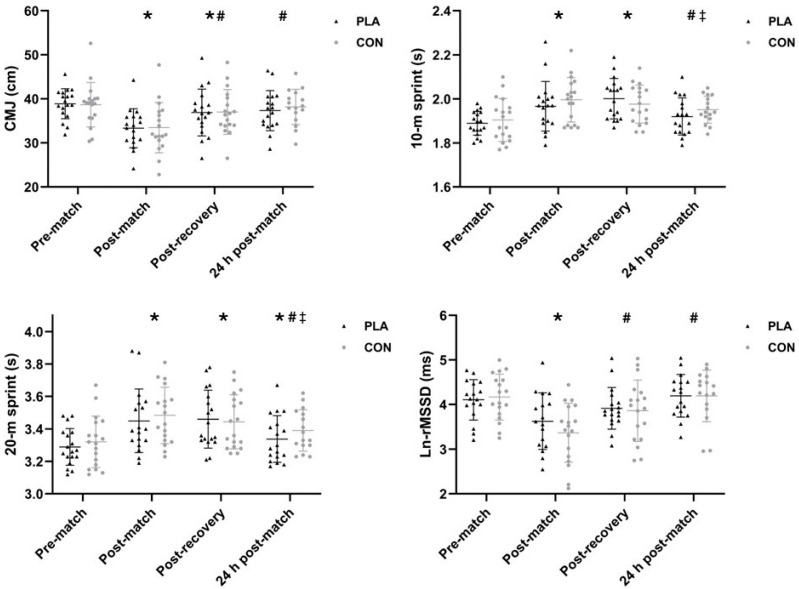
Time course of jump height, 10 m sprint times, 20 m sprint times, and heart rate variability throughout the study period. Data are presented as mean ± standard deviation, with individual values shown in black (PLA) and gray (CON). The pooled effects of time are marked as follows: *, significant difference with pre-match; #, significant difference with post-match; ‡, significant difference with post-recovery. Significant changes shown in the figure were associated with *small-to-large* effect sizes. Statistical significance is set at *p* < 0.05. Abbreviations: CMJ, countermovement jump; Ln-rMSSD, log-transformed squared root of the mean sum of the squared differences between R-R intervals; PLA, placebo intervention; CON, control intervention.

**Figure 4:**
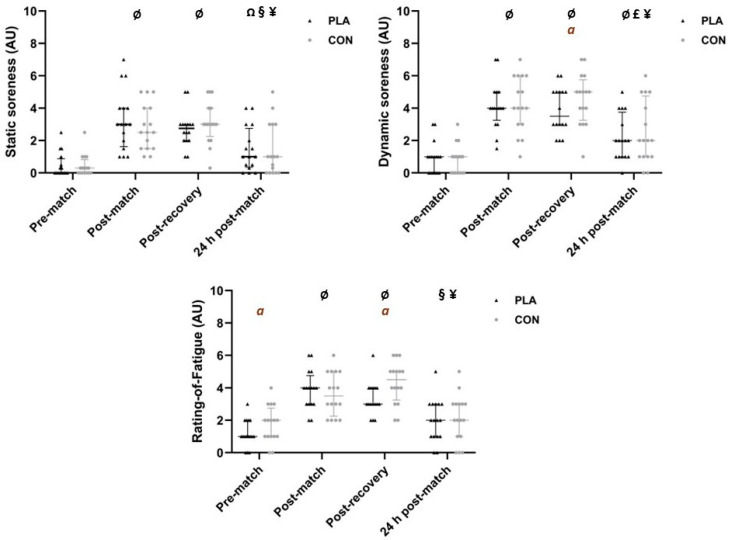
Time course of static muscle soreness, dynamic muscle soreness, and perceived fatigue throughout the study period. Data are presented as median ± interquartile range, with individual values shown in black (PLA) and gray (CON). Ø, significant difference with pre-match in both interventions; Ω, significant difference with pre-match (only in PLA); §, significant difference with post-match in both interventions; ¥, significant difference with post-recovery in both interventions; £, significant difference with post-match (only in PLA); a, *moderate* difference between interventions. Significant changes shown in the figure were associated with *moderate-to-large* effect sizes. Statistical significance is set at *p* < 0.05. Abbreviations: AU, arbitrary units; PLA, placebo intervention; CON, control intervention.

The time course of performance, physiological, and perceptual outcomes throughout the study is shown in Figure 3 and Figure 4. Additionally, individual data for each participant (including percentage change across time points for both interventions) are available in the Supplementary Materials (Tables S2–S8).

No significant effects of time*intervention interaction (CMJ: *p* = 0.850; 10 m: *p* = 0.171; 20 m: *p* = 0.402; Ln-rMSSD: *p* = 0.370) or intervention (CMJ: *p* = 0.623; 10 m: *p* = 0.222; 20 m: *p* = 0.090; Ln-rMSSD: *p* = 0.463) were observed for any of the continuous variables, while significant effects of time (*p* < 0.001) were found for all of them. Post hoc analyses revealed significant worsening (*p* < 0.001) from pre- to post-match in CMJ, 10 m and 20 m sprint, and Ln-rMSSD (ES = −0.83–−1.83, *moderate-to-large*). Similar declines were observed from pre-match to post-recovery in CMJ, 10 m, and 20 m sprint (*p* ≤ 0.002; ES = −0.59–1.14, *small-to-moderate*), with impairments in 20 m sprint persisting at 24 h post-match [*p* = 0.041; ES = 0.63 (95% CI: 0.26–1.00), *moderate*]. Additional differences (*p* ≤ 0.006) were found from post-match to post-recovery in CMJ and Ln-rMSSD (ES = 0.64–1.54, *moderate-to-large*), and from post-match to 24 h post-match in CMJ, 10 m and 20 m sprint, and Ln-rMSSD (ES = −0.67–1.40, *moderate-to-large*). Finally, both 10 m and 20 m sprint times improved significantly at 24 h post-match compared to post-recovery (*p* < 0.001; ES = −0.75–−0.83, *moderate*).

Regarding perceptual data, no significant differences were observed between interventions at corresponding time points (*p* > 0.05). Interestingly, however, ES analysis revealed *moderate* differences between interventions for dynamic soreness at post-recovery (*r* = 0.32), and for perceived fatigue at both pre-match (*r* = 0.34) and post-recovery (*r* = 0.40), indicating higher well-being in PLA. Additionally, a significant effect of time (*p* < 0.001) was found within each intervention for all variables. Specifically—compared to pre-match—static soreness was higher at post-match (*p* = 0.004, *r* = 0.60, *large*) and post-recovery (*p* = 0.015–0.046, *r* = 0.62, *large*) in both interventions, and at 24 h compared to pre-match in PLA only (*p* = 0.037, *r* = 0.49, *moderate*). Furthermore, static soreness was lower at 24 h compared to post-match in both PLA and CON (*p* = 0.014–0.024, *r* = 0.51–0.54, *large*). For both interventions, dynamic soreness was higher at all time points compared to pre-match (*p* = 0.002–0.047, *r* = 0.48–0.63, *moderate-to-large*), and lower at 24 h post-match compared to post-recovery (*p* = 0.007–0.043, *r* = 0.48–0.58, *moderate-to-large*). Lower values were observed at 24 h post-match, compared to post-match, in PLA only (*p* = 0.011, *r* = 0.56, *large*). Finally, in both interventions, perceived fatigue was higher at post-match and post-recovery compared to pre-match (*p* = 0.002–0.004, *r* = 0.61–0.63, *large*), and lower at 24 h post-match compared to post-match and post-recovery (*p* = 0.004–0.010, *r* = 0.56–0.62, *large*).

## 4. Discussion

Our main findings indicate that the use of a post-match placebo intervention did not produce substantial benefits in terms of performance and parasympathetic reactivation following the football match. Conversely, *moderate* improvements were found for dynamic soreness and perceived fatigue shortly after the placebo intervention (i.e., post-recovery), compared to passive rest.

Recent evidence has suggested that the placebo effect can have a sizeable impact on performance-related measures in sport. Specifically, data from two systematic reviews [8,9] indicate that placebo interventions (especially when presented as anabolic steroids, transcutaneous nerve stimulation, or caffeine) produced overall *moderate* effects (ranging from *small* to *very large*) on performance outcomes (i.e., cycling/running time trial performance, strength and power measures). Similarly, one of the very few studies on recovery strategies found that a fruit-flavored placebo drink attenuated impairments in 10 m sprint and repeated sprint ability 24 h after a football-specific fatiguing protocol, compared to passive rest [5]. Despite these findings, our analyses revealed non-significant, *trivial-to-small* differences between PLA and CON, suggesting that the placebo intervention did not lead to substantial performance improvements. Crucially, previous research has highlighted that the potential benefits of a placebo may depend on the athlete’s belief in its effectiveness [9]. Accordingly, participants in Nasser et al. [5] showed significantly stronger belief in the placebo intervention compared to passive rest, which may explain their positive findings. In support of this hypothesis, Borne et al. [11] reported comparable belief ratings for sham electrostimulation (placebo) and passive rest, which may explain the absence of significant, between-intervention differences in peak or mean power output during recovery from repeated Wingate tests. As belief in the effectiveness of vagus nerve stimulation was not assessed in the present study, it remains unknown whether any differences in expectation existed between PLA and CON, and how these may have influenced our results.

In addition to performance decreases, exercise-induced fatigue can suppress parasympathetic activity, reflecting changes in cardiovascular homeostasis that can negatively affect recovery [26]. Crucially, previous research suggests that placebo effects could influence HRV, as the expectation of effective treatment may engage brain regions that regulate autonomic activity [27]. Despite these considerations, the impact of the placebo effect on HRV recovery has been rarely investigated. A study on recovery from psychosocial stress—induced by two mental arithmetic tasks—found that an intranasal placebo presented as serotonin led to improvements in HRV after the second task compared to the first, relative to control [28]. Conversely, our data revealed non-significant, *trivial-to-small* differences between PLA and CON at corresponding time points. As described for performance-related outcomes, it is possible that insufficient belief in the placebo intervention prevented a placebo effect from emerging. Furthermore, the return of HRV to baseline at post-recovery suggests that parasympathetic reactivation had already been achieved, making it difficult to detect potential differences between PLA and CON at that stage. Finally, methodological differences (e.g., the type of stressor, placebo intervention, sample characteristics) between our study and previous research [28] limit the possibility of direct comparisons. Given the limited research in this area, further studies are needed before confident conclusions can be drawn.

Even when recovery interventions show limited performance or physiological effects, they may still support perceptual recovery [29]. The use of placebo-based recovery strategies fits well with this aim, as positive expectations about their effectiveness can influence psychological components of recovery [27]. Despite the lack of statistically significant between-intervention differences in our study, the placebo effect observed through effect size analysis holds practical relevance, as such potential benefits are meaningful for athletes [9]. Accordingly, our findings revealed *moderately* lower levels of dynamic muscle soreness and perceived fatigue at post-recovery in PLA compared to CON. These results align with Nasser et al. [5], who found that the consumption of a placebo beverage after a football-specific fatiguing protocol led to reduced muscle soreness compared to control. Such findings have important implications for both research and practice. First, since studies often report improvements in perceptual outcomes (e.g., muscle soreness, perceived fatigue) without corresponding changes in performance or physiological measures [12], isolating placebo effects from intervention-specific effects appears important to better understand what contributes to effective recovery. Second, from a practical standpoint, these findings suggest that athletes’ belief in the effectiveness of a recovery strategy may benefit perceptual recovery. This should not be understated, as improving athletes’ well-being can play an important role in supporting psychological recovery [29]. For full transparency, it should be acknowledged that not all perceptual outcomes varied substantially between interventions, as static soreness only showed *trivial-to-small* differences between PLA and CON at corresponding time points. Additionally, perceived fatigue was *moderately* lower in PLA than CON at pre-match, indicating a baseline imbalance. Although fatigue scores were similar between conditions at post-match (which would suggest that players began the recovery intervention in comparable states), the post-recovery difference might have been partly influenced by the differences observed at baseline. Nonetheless, considering the overall findings from the present study, combined with those from Nasser et al. [5], we stand by the interpretation that placebo effects should be considered when designing studies on recovery interventions and when utilizing placebos in applied sport settings.

Although the present study expands current knowledge regarding the placebo effect in recovery strategies, some limitations should be acknowledged. First, the participants were male youth football players, which may limit the applicability of our findings to athletes of different sex, age, competitive level, or sporting context. Second, belief in the effectiveness of the placebo intervention was not assessed, which partly limits the possibility of determining whether expectancy influenced the observed outcomes (especially performance-related measures and HRV). Finally, our design compared a placebo intervention with passive rest, as the aim of this study was to isolate the placebo effect. However, including a third condition (i.e., an actual recovery strategy) could allow to separate this phenomenon from the “true” physiological effects of a given intervention, offering a more complete understanding of how expectancy contributes to recovery outcomes.

## 5. Conclusions

The present findings indicate that a post-exercise placebo intervention (presented as vagus nerve stimulation) did not substantially improve performance or cardiac parasympathetic reactivation following a football match in youth male players. However, *moderate* improvements in dynamic muscle soreness and perceived fatigue at post-recovery (compared to passive rest) suggest that placebo-related mechanisms may benefit perceptual aspects of recovery. Hence, this study provides specific evidence that placebo-induced improvements in subjective recovery can occur independently of performance-related or physiological changes under ecologically valid conditions.

These findings have important implications for both research and practice. Specifically, in research settings, using a placebo instead of passive rest as the control condition may offer a more appropriate comparison, while including all three conditions (i.e., the recovery strategy itself, placebo, and passive rest) could help separate psychological from physiological effects. From a practical perspective, coaching staff members may consider boosting athletes’ expectations around recovery strategies to make them feel more effective and support psychological recovery, even where benefits on physiological or performance measures are limited.

## Figures and Tables

**Table 1:** Differences in external and internal load between interventions.

Load Variables	PLA	CON	*p* Value	ES (95% CI)	Interpretation
PL (AU)	126.9 ± 12.2	126.0 ± 13.9	0.649	0.13 (−0.42–0.67)	*Trivial*
PL/min (AU/min)	1.34 ± 0.13	1.32 ± 0.15	0.631	0.14 (−0.41–0.68)	*Trivial*
Total distance (m)	10,476 ± 760	10,484 ± 758	0.950	−0.02 (0.56–0.53)	*Trivial*
%HR_peak_	84.1 ± 3.2	83.7 ± 2.9	0.705	0.12 (−0.48–0.71)	*Trivial*
s-RPE (AU) *	385.3 ± 147.4	374.7 ± 110.4	0.797	0.05 (N/A)	*Trivial*

* A Wilcoxon test was used for non-normally distributed s-RPE data, with effect size calculated as Wilcoxon’s *r* (z/√N) instead of Cohen’s *d*. Abbreviations: PL, Player Load; PL/min, Player Load per minute; %HR_peak_, average session heart rate, measured as the percentage of peak heart rate; s-RPE, session-Rating of Perceived Exertion; AU, arbitrary units; PLA, placebo intervention; CON, control intervention; ES, effect size; CI, confidence interval; N/A, not available.

## Data Availability

The dataset associated with this study is included in the Supplementary Materials. Further inquiries can be directed to the corresponding author.

## Supplementary Materials

The following supporting information can be downloaded at https://www.mdpi.com/article/10.3390/healthcare14010004/s1; Table S1: Warm-up protocol; Table S2: Individual data and % change in countermovement jump height (cm) across time points; Table S3: Individual data and % change in 10 m sprint times (s) across time points; Table S4: Individual data and % change in 20 m sprint times (s) across time points; Table S5: Individual data and % change in Ln-rMSSD (ms) across time points; Table S6: Individual data and % change in static muscle soreness (arbitrary units) across time points; Table S7: Individual data and % change in dynamic muscle soreness (arbitrary units) across time points; Table S8: Individual data and % change in perceived fatigue (arbitrary units) across time points.
