# Supplementary material for: The Power of Belief: Investigating the Placebo Effect in Post-Exercise Recovery Strategies for Football Players
Source: Healthcare (Basel). 2025 Dec 19;14(1):4. doi: 10.3390/healthcare14010004 (PMC12785754; doi:10.3390/healthcare14010004)
Supplement: Supplementary file 1 [file healthcare-14-00004-s001.zip › healthcare-4015251-SI.pdf]

SUPPLEMENTARY MATERIAL

Table S1. Warm-up protocol

	Exercise	Repetitions	Rep duration	Recovery
**Raise (4’30”)**	Jogging	1	30”	-
	Carioca	1	15”	15” jogging
	Side shuffle	1	15”	15” jogging
	Backward jogging	1	15”	15” jogging
	Alternate backward/forward jogging	1	15”	15” jogging
	High skips	1	15”	15” jogging
	High knees	1	15”	15” jogging
	A-skips	1	15”	15” jogging
	Butt kicks	1	15”	15” jogging
**Activate/mobilize (2’30”)**	Front-to-back leg swings	1	30”	-
	Side-to-side leg swings	1	30”	-
	Jog with knee-to-chest	1	30”	-
	Squat and jog	1	30”	-
	Walking lunges + trunk rotation	1	30”	-
**Potentiate (3’)**	Jog-to-sprint progression	2	10”	20” jogging
	Jog-stop-sprint	2	10”	20” jogging
	Turn-and-sprint	2	10”	20” jogging

Table S2. Individual data and % change in **countermovement jump height (cm)** across time points

ID	Intervention	PRE	POST	POST REC	24H	% change PRE-POST	% change PRE-POST REC	% change PRE-24H	% change POST-POST REC	% change POST-24H	% change POST REC-24H
Player 1	PLA	45.6	44.3	49.3	46.4	-2.85	8.11	1.75	11.29	4.74	-5.88
Player 2	PLA	42.0	34.3	35.6	35.0	-18.33	-15.24	-16.67	3.79	2.04	-1.69
Player 3	PLA	40.7	37.4	40.8	39.1	-8.11	0.25	-3.93	9.09	4.55	-4.17
Player 4	PLA	34.3	29.0	33.9	33.7	-15.45	-1.17	-1.75	16.90	16.21	-0.59
Player 5	PLA	38.4	33.4	38.2	38.0	-13.02	-0.52	-1.04	14.37	13.77	-0.52
Player 6	PLA	33.5	28.2	30.4	31.5	-15.82	-9.25	-5.97	7.80	11.70	3.62
Player 7	PLA	36.9	34.0	34.7	34.4	-7.86	-5.96	-6.78	2.06	1.18	-0.86
Player 8	PLA	41.4	36.0	43.0	40.5	-13.04	3.86	-2.17	19.44	12.50	-5.81
Player 9	PLA	40.5	37.1	39.0	38.9	-8.40	-3.70	-3.95	5.12	4.85	-0.26
Player 10	PLA	41.5	35.9	38.6	45.8	-13.49	-6.99	10.36	7.52	27.58	18.65
Player 11	PLA	38.6	30.6	36.9	36.9	-20.73	-4.40	-4.40	20.59	20.59	0.00
Player 12	PLA	42.1	35.9	43.7	40.7	-14.73	3.80	-3.33	21.73	13.37	-6.86
Player 13	PLA	31.9	24.2	26.5	28.6	-24.14	-16.93	-10.34	9.50	18.18	7.92
Player 14	PLA	37.9	32.8	37.5	36.1	-13.46	-1.06	-4.75	14.33	10.06	-3.73
Player 15	PLA	38.6	30.8	36.7	37.9	-20.21	-4.92	-1.81	19.16	23.05	3.27
Player 16	PLA	40.5	31.3	32.5	40.5	-22.72	-19.75	0.00	3.83	29.39	24.62
Player 17	PLA	36.1	34.7	35.9	34.2	-3.88	-0.55	-5.26	3.46	-1.44	-4.74
Player 18	PLA	39.5	30.2	31.1	34.0	-23.54	-21.27	-13.92	2.98	12.58	9.32
Player 1	CON	52.6	47.7	48.3	45.8	-9.32	-8.17	-12.93	1.26	-3.98	-5.18
Player 2	CON	38.7	37.4	38.6	40.1	-3.36	-0.26	3.62	3.21	7.22	3.89
Player 3	CON	41.8	37.1	40.2	39.1	-11.24	-3.83	-6.46	8.36	5.39	-2.74
Player 4	CON	36.4	25.8	32.8	33.8	-29.12	-9.89	-7.14	27.13	31.01	3.05
Player 5	CON	39.1	33.9	34.2	36.9	-13.30	-12.53	-5.63	0.88	8.85	7.89
Player 6	CON	30.8	31.4	35.2	35.2	1.95	14.29	14.29	12.10	12.10	0.00
Player 7	CON	38.9	32.0	33.9	-	-17.74	-12.85	-	5.94	-	-
Player 8	CON	40.1	31.9	34.2	38.4	-20.45	-14.71	-4.24	7.21	20.38	12.28
Player 9	CON	39.1	35.1	36.7	37.9	-10.23	-6.14	-3.07	4.56	7.98	3.27
Player 10	CON	44.7	39.0	43.0	41.4	-12.75	-3.80	-7.38	10.26	6.15	-3.72
Player 11	CON	39.4	29.3	37.2	37.5	-25.63	-5.58	-4.82	26.96	27.99	0.81
Player 12	CON	40.0	40.4	44.7	42.5	1.00	11.75	6.25	10.64	5.20	-4.92
Player 13	CON	30.4	22.8	26.5	29.7	-25.00	-12.83	-2.30	16.23	30.26	12.08
Player 14	CON	35.6	30.6	34.0	-	-14.04	-4.49	-	11.11	-	-
Player 15	CON	40.0	36.3	41.0	42.0	-9.25	2.50	5.00	12.95	15.70	2.44
Player 16	CON	39.5	28.7	37.1	39.8	-27.34	-6.08	0.76	29.27	38.68	7.28
Player 17	CON	35.7	31.5	36.4	37.9	-11.76	1.96	6.16	15.56	20.32	4.12
Player 18	CON	33.3	31.5	32.4	32.8	-5.41	-2.70	-1.50	2.86	4.13	1.23

Table S3. Individual data and % change in **10-m sprint times (s)** across time points

ID	Intervention	PRE	POST	POST REC	24H	% change PRE-POST	% change PRE-POST REC	% change PRE-24H	% change POST-POST REC	% change POST-24H	% change POST REC-24H
Player 1	PLA	1.96	2.01	2.05	2.03	2.55	4.59	3.57	1.99	1.00	-0.98
Player 2	PLA	1.87	1.97	1.99	1.85	5.35	6.42	-1.07	1.02	-6.09	-7.04
Player 3	PLA	1.86	1.97	1.91	1.93	5.91	2.69	3.76	-3.05	-2.03	1.05
Player 4	PLA	1.90	1.97	1.94	1.96	3.68	2.11	3.16	-1.52	-0.51	1.03
Player 5	PLA	1.91	1.83	1.87	1.89	-4.19	-2.09	-1.05	2.19	3.28	1.07
Player 6	PLA	1.94	2.16	2.14	2.10	11.34	10.31	8.25	-0.93	-2.78	-1.87
Player 7	PLA	1.82	1.85	1.90	1.85	1.65	4.40	1.65	2.70	0.00	-2.63
Player 8	PLA	1.90	2.00	1.90	1.89	5.26	0.00	-0.53	-5.00	-5.50	-0.53
Player 9	PLA	1.95	1.79	1.95	1.82	-8.21	0.00	-6.67	8.94	1.68	-6.67
Player 10	PLA	1.80	1.89	2.04	1.79	5.00	13.33	-0.56	7.94	-5.29	-12.25
Player 11	PLA	1.98	2.26	2.19	2.02	14.14	10.61	2.02	-3.10	-10.62	-7.76
Player 12	PLA	1.81	1.90	1.93	1.88	4.97	6.63	3.87	1.58	-1.05	-2.59
Player 13	PLA	1.93	2.01	2.05	2.02	4.15	6.22	4.66	1.99	0.50	-1.46
Player 14	PLA	1.86	1.91	2.04	1.93	2.69	9.68	3.76	6.81	1.05	-5.39
Player 15	PLA	1.84	1.89	1.91	1.84	2.72	3.80	0.00	1.06	-2.65	-3.66
Player 16	PLA	1.86	2.03	2.10	1.85	9.14	12.90	-0.54	3.45	-8.87	-11.90
Player 17	PLA	1.94	1.99	2.04	1.98	2.58	5.15	2.06	2.51	-0.50	-2.94
Player 18	PLA	1.87	1.96	2.07	1.93	4.81	10.70	3.21	5.61	-1.53	-6.76
Player 1	CON	1.87	1.87	1.95	1.93	0.00	4.28	3.21	4.28	3.21	-1.03
Player 2	CON	1.94	1.98	1.90	1.94	2.06	-2.06	0.00	-4.04	-2.02	2.11
Player 3	CON	1.83	1.88	1.85	1.87	2.73	1.09	2.19	-1.60	-0.53	1.08
Player 4	CON	1.83	2.02	2.08	1.96	10.38	13.66	7.10	2.97	-2.97	-5.77
Player 5	CON	1.78	1.88	1.98	1.92	5.62	11.24	7.87	5.32	2.13	-3.03
Player 6	CON	2.10	2.22	2.14	2.05	5.71	1.90	-2.38	-3.60	-7.66	-4.21
Player 7	CON	1.77	1.87	1.88	-	5.65	6.21	-	0.53	-	-
Player 8	CON	1.84	1.98	1.89	1.84	7.61	2.72	0.00	-4.55	-7.07	-2.65
Player 9	CON	1.81	2.00	1.94	1.94	10.50	7.18	7.18	-3.00	-3.00	0.00
Player 10	CON	1.96	2.03	2.01	2.02	3.57	2.55	3.06	-0.99	-0.49	0.50
Player 11	CON	1.87	2.10	2.05	2.03	12.30	9.63	8.56	-2.38	-3.33	-0.98
Player 12	CON	1.92	1.92	1.91	1.91	0.00	-0.52	-0.52	-0.52	-0.52	0.00
Player 13	CON	2.01	2.03	2.07	2.00	1.00	2.99	-0.50	1.97	-1.48	-3.38
Player 14	CON	1.94	2.07	2.05	-	6.70	5.67	-	-0.97	-	-
Player 15	CON	1.80	1.87	1.85	1.88	3.89	2.78	4.44	-1.07	0.53	1.62
Player 16	CON	1.95	2.12	2.04	1.98	8.72	4.62	1.54	-3.77	-6.60	-2.94
Player 17	CON	2.06	2.01	1.95	1.93	-2.43	-5.34	-6.31	-2.99	-3.98	-1.03
Player 18	CON	2.00	2.08	2.03	2.02	4.00	1.50	1.00	-2.40	-2.88	-0.49

Table S4. Individual data and % change in **20-m sprint times (s)** across time points

ID	Intervention	PRE	POST	POST REC	24H	% change PRE-POST	% change PRE-POST REC	% change PRE-24H	% change POST-POST REC	% change POST-24H	% change POST REC-24H
Player 1	PLA	3.26	3.38	3.33	3.37	3.68	2.15	3.37	-1.48	-0.30	1.20
Player 2	PLA	3.25	3.52	3.52	3.28	8.31	8.31	0.92	0.00	-6.82	-6.82
Player 3	PLA	3.23	3.41	3.32	3.39	5.57	2.79	4.95	-2.64	-0.59	2.11
Player 4	PLA	3.38	3.56	3.36	3.41	5.33	-0.59	0.89	-5.62	-4.21	1.49
Player 5	PLA	3.36	3.27	3.32	3.24	-2.68	-1.19	-3.57	1.53	-0.92	-2.41
Player 6	PLA	3.48	3.87	3.78	3.67	11.21	8.62	5.46	-2.33	-5.17	-2.91
Player 7	PLA	3.15	3.22	3.22	3.19	2.22	2.22	1.27	0.00	-0.93	-0.93
Player 8	PLA	3.23	3.4	3.21	3.23	5.26	-0.62	0.00	-5.59	-5.00	0.62
Player 9	PLA	3.29	3.19	3.35	3.18	-3.04	1.82	-3.34	5.02	-0.31	-5.07
Player 10	PLA	3.14	3.33	3.44	3.17	6.05	9.55	0.96	3.30	-4.80	-7.85
Player 11	PLA	3.46	3.88	3.76	3.51	12.14	8.67	1.45	-3.09	-9.54	-6.65
Player 12	PLA	3.12	3.30	3.34	3.24	5.77	7.05	3.85	1.21	-1.82	-2.99
Player 13	PLA	3.38	3.34	3.60	3.51	-1.18	6.51	3.85	7.78	5.09	-2.50
Player 14	PLA	3.23	3.33	3.50	3.30	3.10	8.36	2.17	5.11	-0.90	-5.71
Player 15	PLA	3.19	3.39	3.34	3.20	6.27	4.70	0.31	-1.47	-5.60	-4.19
Player 16	PLA	3.31	3.61	3.67	3.26	9.06	10.88	-1.51	1.66	-9.70	-11.17
Player 17	PLA	3.48	3.53	3.56	3.5	1.44	2.30	0.57	0.85	-0.85	-1.69
Player 18	PLA	3.27	3.57	3.66	3.43	9.17	11.93	4.89	2.52	-3.92	-6.28
Player 1	CON	3.21	3.26	3.25	3.23	1.56	1.25	0.62	-0.31	-0.92	-0.62
Player 2	CON	3.32	3.5	3.34	3.3	5.42	0.60	-0.60	-4.57	-5.71	-1.20
Player 3	CON	3.28	3.33	3.32	3.33	1.52	1.22	1.52	-0.30	0.00	0.30
Player 4	CON	3.25	3.54	3.56	3.41	8.92	9.54	4.92	0.56	-3.67	-4.21
Player 5	CON	3.15	3.39	3.45	3.33	7.62	9.52	5.71	1.77	-1.77	-3.48
Player 6	CON	3.67	3.81	3.75	3.62	3.81	2.18	-1.36	-1.57	-4.99	-3.47
Player 7	CON	3.13	3.23	3.25	-	3.19	3.83	-	0.62	-	-
Player 8	CON	3.12	3.43	3.29	3.23	9.94	5.45	3.53	-4.08	-5.83	-1.82
Player 9	CON	3.15	3.36	3.32	3.3	6.67	5.40	4.76	-1.19	-1.79	-0.60
Player 10	CON	3.34	3.41	3.36	3.38	2.10	0.60	1.20	-1.47	-0.88	0.60
Player 11	CON	3.36	3.71	3.58	3.58	10.42	6.55	6.55	-3.50	-3.50	0.00
Player 12	CON	3.31	3.32	3.28	3.31	0.30	-0.91	0.00	-1.20	-0.30	0.91
Player 13	CON	3.44	3.58	3.7	3.49	4.07	7.56	1.45	3.35	-2.51	-5.68
Player 14	CON	3.36	3.56	3.54	-	5.95	5.36	-	-0.56	-	-
Player 15	CON	3.18	3.3	3.28	3.24	3.77	3.14	1.89	-0.61	-1.82	-1.22
Player 16	CON	3.45	3.68	3.62	3.46	6.67	4.93	0.29	-1.63	-5.98	-4.42
Player 17	CON	3.59	3.57	3.46	3.51	-0.56	-3.62	-2.23	-3.08	-1.68	1.45
Player 18	CON	3.47	3.72	3.64	3.53	7.20	4.90	1.73	-2.15	-5.11	-3.02

Table S5. Individual data and % change in **Ln-rMSSD (ms)** across time points

ID	Intervention	PRE	POST	POST REC	24H	% change PRE-POST	% change PRE-POST REC	% change PRE-24H	% change POST-POST REC	% change POST-24H	% change POST REC-24H
Player 1	PLA	4.77	4.94	5.04	4.66	3.56	5.66	-2.31	2.02	-5.67	-7.54
Player 2	PLA	4.23	3.46	3.08	4.50	-18.20	-27.19	6.38	-10.98	30.06	46.10
Player 3	PLA	4.47	3.23	3.64	4.33	-27.74	-18.57	-3.13	12.69	34.06	18.96
Player 4	PLA	4.00	2.55	3.58	3.57	-36.25	-10.50	-10.75	40.39	40.00	-0.28
Player 5	PLA	3.21	3.98	3.86	3.85	23.99	20.25	19.94	-3.02	-3.27	-0.26
Player 6	PLA	4.03	3.91	3.90	4.06	-2.98	-3.23	0.74	-0.26	3.84	4.10
Player 7	PLA	3.46	3.10	3.84	3.95	-10.40	10.98	14.16	23.87	27.42	2.86
Player 8	PLA	3.35	3.62	4.04	3.96	8.06	20.60	18.21	11.60	9.39	-1.98
Player 9	PLA	4.51	3.71	3.93	5.05	-17.74	-12.86	11.97	5.93	36.12	28.50
Player 10	PLA	3.79	-	4.22	3.79	-	11.35	0.00	-	-	-10.19
Player 11	PLA	4.11	2.96	3.30	3.27	-27.98	-19.71	-20.44	11.49	10.47	-0.91
Player 12	PLA	-	3.07	3.66	-	-	-	-	19.22	-	-
Player 13	PLA	4.71	3.56	4.33	4.90	-24.42	-8.07	4.03	21.63	37.64	13.16
Player 14	PLA	3.98	4.25	3.75	4.37	6.78	-5.78	9.80	-11.76	2.82	16.53
Player 15	PLA	4.54	2.80	3.78	4.28	-38.33	-16.74	-5.73	35.00	52.86	13.23
Player 16	PLA	4.13	4.02	4.01	3.75	-2.66	-2.91	-9.20	-0.25	-6.72	-6.48
Player 17	PLA	4.24	4.25	3.77	4.52	0.24	-11.08	6.60	-11.29	6.35	19.89
Player 18	PLA	4.29	4.29	4.77	4.51	0.00	11.19	5.13	11.19	5.13	-5.45
Player 1	CON	4.72	3.02	5.03	3.90	-36.02	6.57	-17.37	66.56	29.14	-22.47
Player 2	CON	3.58	3.31	3.86	4.26	-7.54	7.82	18.99	16.62	28.70	10.36
Player 3	CON	4.01	4.10	3.52	4.24	2.24	-12.22	5.74	-14.15	3.41	20.45
Player 4	CON	3.79	2.21	3.04	2.96	-41.69	-19.79	-21.90	37.56	33.94	-2.63
Player 5	CON	3.99	3.60	3.22	3.70	-9.77	-19.30	-7.27	-10.56	2.78	14.91
Player 6	CON	4.03	4.10	4.34	4.52	1.74	7.69	12.16	5.85	10.24	4.15
Player 7	CON	4.28	2.12	2.77	-	-50.47	-35.28	-	30.66	-	-
Player 8	CON	4.30	3.43	2.75	4.01	-20.23	-36.05	-6.74	-19.83	16.91	45.82
Player 9	CON	5.00	4.44	4.27	4.66	-11.20	-14.60	-6.80	-3.83	4.95	9.13
Player 10	CON	3.36	3.63	4.10	4.21	8.04	22.02	25.30	12.95	15.98	2.68
Player 11	CON	4.55	2.64	3.22	4.90	-41.98	-29.23	7.69	21.97	85.61	52.17
Player 12	CON	3.25	2.84	3.58	2.97	-12.62	10.15	-8.62	26.06	4.58	-17.04
Player 13	CON	4.75	3.63	4.78	4.81	-23.58	0.63	1.26	31.68	32.51	0.63
Player 14	CON	3.70	3.98	3.98	-	7.57	7.57	-	0.00	-	-
Player 15	CON	4.80	3.41	4.10	4.63	-28.96	-14.58	-3.54	20.23	35.78	12.93
Player 16	CON	3.94	3.52	3.91	4.43	-10.66	-0.76	12.44	11.08	25.85	13.30
Player 17	CON	4.44	2.73	4.14	4.42	-38.51	-6.76	-0.45	51.65	61.90	6.76
Player 18	CON	4.54	3.95	4.89	4.49	-13.00	7.71	-1.10	23.80	13.67	-8.18

Table S6. Individual data and % change in **static muscle soreness (arbitrary units)** across time points

ID	Intervention	PRE	POST	POST REC	24H	% change PRE-POST	% change PRE-POST REC	% change PRE-24H	% change POST-POST REC	% change POST-24H	% change POST REC-24H
Player 1	PLA	0	4	2.5	0	-	-	-	-37.50	-100.00	-100.00
Player 2	PLA	2.5	2.5	3	3	0.00	20.00	20.00	20.00	20.00	0.00
Player 3	PLA	0.3	1.5	1	0	400.00	233.33	-100.00	-33.33	-100.00	-100.00
Player 4	PLA	1	3	2.5	0.5	200.00	150.00	-50.00	-16.67	-83.33	-80.00
Player 5	PLA	0	1	2	0.5	-	-	-	100.00	-50.00	-75.00
Player 6	PLA	0	7	5	4	-	-	-	-28.57	-42.86	-20.00
Player 8	PLA	1.5	2	2	2	33.33	33.33	33.33	0.00	0.00	0.00
Player 9	PLA	0.3	6	5	3	1900.00	1566.67	900.00	-16.67	-50.00	-40.00
Player 10	PLA	0	1	1	0	-	-	-	0.00	-100.00	-100.00
Player 11	PLA	0	3	3	0.3	-	-	-	0.00	-90.00	-90.00
Player 12	PLA	0.5	4	2	4	700.00	300.00	700.00	-50.00	0.00	100.00
Player 13	PLA	0	6	3	1.5	-	-	-	-50.00	-75.00	-50.00
Player 15	PLA	0	3	2.5	1	-	-	-	-16.67	-66.67	-60.00
Player 16	PLA	0	4	3	1	-	-	-	-25.00	-75.00	-66.67
Player 17	PLA	1.5	3	3	1.5	100.00	100.00	0.00	0.00	-50.00	-50.00
Player 18	PLA	0	1	3	1	-	-	-	200.00	0.00	-66.67
Player 1	CON	0	1	1.5	0	-	-	-	50.00	-100.00	-100.00
Player 2	CON	2.5	5	5	5	100.00	100.00	100.00	0.00	0.00	0.00
Player 3	CON	0	2	3	3	-	-	-	50.00	50.00	0.00
Player 4	CON	0	4	4	0.5	-	-	-	0.00	-87.50	-87.50
Player 5	CON	0	4	3	1	-	-	-	-25.00	-75.00	-66.67
Player 6	CON	0	1.5	3	0	-	-	-	100.00	-100.00	-100.00
Player 8	CON	0.3	3	3	3	900.00	900.00	900.00	0.00	0.00	0.00
Player 9	CON	0.3	5	4	1	1566.67	1233.33	233.33	-20.00	-80.00	-75.00
Player 10	CON	0.3	2.5	2	0	733.33	566.67	-100.00	-20.00	-100.00	-100.00
Player 11	CON	0	1.5	0.3	0	-	-	-	-80.00	-100.00	-100.00
Player 12	CON	1	5	5	4	400.00	400.00	300.00	0.00	-20.00	-20.00
Player 13	CON	0.3	3	3	0.3	900.00	900.00	0.00	0.00	-90.00	-90.00
Player 15	CON	1	1	2	1	0.00	100.00	0.00	100.00	0.00	-50.00
Player 16	CON	0	2.5	5	1	-	-	-	100.00	-60.00	-80.00
Player 17	CON	1	2.5	3	3	150.00	200.00	200.00	20.00	20.00	0.00
Player 18	CON	0.3	1.5	3	0	400.00	900.00	-100.00	100.00	-100.00	-100.00

Table S7. Individual data and % change in **dynamic muscle soreness (arbitrary units)** across time points

ID	Intervention	PRE	POST	POST REC	24H	% change PRE-POST	% change PRE-POST REC	% change PRE-24H	% change POST-POST REC	% change POST-24H	% change POST REC-24H
Player 1	PLA	1	5	3	1	400.00	200.00	0.00	-40.00	-80.00	-66.67
Player 2	PLA	3	4	6	5	33.33	100.00	66.67	50.00	25.00	-16.67
Player 3	PLA	0	4	2	0	-	-	-	-50.00	-100.00	-100.00
Player 4	PLA	1	4	3	1	300.00	200.00	0.00	-25.00	-75.00	-66.67
Player 5	PLA	1	2	2	1	100.00	100.00	0.00	0.00	-50.00	-50.00
Player 6	PLA	0	7	6	4	-	-	-	-14.29	-42.86	-33.33
Player 8	PLA	3	4	4	2	33.33	33.33	-33.33	0.00	-50.00	-50.00
Player 9	PLA	1	7	5	4	600.00	400.00	300.00	-28.57	-42.86	-20.00
Player 10	PLA	0	3	2	2	-	-	-	-33.33	-33.33	0.00
Player 11	PLA	0	5	5	1	-	-	-	0.00	-80.00	-80.00
Player 12	PLA	1	4	3	4	300.00	200.00	300.00	-25.00	0.00	33.33
Player 13	PLA	1	2	5	2	100.00	400.00	100.00	150.00	0.00	-60.00
Player 15	PLA	1	4	3	2	300.00	200.00	100.00	-25.00	-50.00	-33.33
Player 16	PLA	0	4	5	3	-	-	-	25.00	-25.00	-40.00
Player 17	PLA	2	5	5	2	150.00	150.00	0.00	0.00	-60.00	-60.00
Player 18	PLA	0	3	3	1	-	-	-	0.00	-66.67	-66.67
Player 1	CON	1	3	4	1	200.00	300.00	0.00	33.33	-66.67	-75.00
Player 2	CON	3	6	7	6	100.00	133.33	100.00	16.67	0.00	-14.29
Player 3	CON	0	1	1	5	-	-	-	0.00	400.00	400.00
Player 4	CON	0	7	7	2	-	-	-	0.00	-71.43	-71.43
Player 5	CON	0	5	4	1	-	-	-	-20.00	-80.00	-75.00
Player 6	CON	0	4	3	0	-	-	-	-25.00	-100.00	-100.00
Player 8	CON	1	4	5	4	300.00	400.00	300.00	25.00	0.00	-20.00
Player 9	CON	1	6	5	3	500.00	400.00	200.00	-16.67	-50.00	-40.00
Player 10	CON	1	3	3	1	200.00	200.00	0.00	0.00	-66.67	-66.67
Player 11	CON	0	4	5	1	-	-	-	25.00	-75.00	-80.00
Player 12	CON	1	6	6	5	500.00	500.00	400.00	0.00	-16.67	-16.67
Player 13	CON	2	5	5	2	150.00	150.00	0.00	0.00	-60.00	-60.00
Player 15	CON	1	2	3	2	100.00	200.00	100.00	50.00	0.00	-33.33
Player 16	CON	0	4	6	2	-	-	-	50.00	-50.00	-66.67
Player 17	CON	2	6	5	5	200.00	150.00	150.00	-16.67	-16.67	0.00
Player 18	CON	0	2	4	0	-	-	-	100.00	-100.00	-100.00

Table S8. Individual data and % change in **perceived fatigue (arbitrary units)** across time points

ID	Intervention	PRE	POST	POST REC	24H	% change PRE-POST	% change PRE-POST REC	% change PRE-24H	% change POST-POST REC	% change POST-24H	% change POST REC-24H
Player 1	PLA	1	3	2	1	200.00	100.00	0.00	-33.33	-66.67	-50.00
Player 2	PLA	2	5	6	5	150.00	200.00	150.00	20.00	0.00	-16.67
Player 3	PLA	0	4	2	0	-	-	-	-50.00	-100.00	-100.00
Player 4	PLA	1	3	3	1	200.00	200.00	0.00	0.00	-66.67	-66.67
Player 5	PLA	2	4	3	3	100.00	50.00	50.00	-25.00	-25.00	0.00
Player 6	PLA	1	4	3	0	300.00	200.00	-100.00	-25.00	-100.00	-100.00
Player 8	PLA	3	4	3	3	33.33	0.00	0.00	-25.00	-25.00	0.00
Player 9	PLA	1	3	4	1	200.00	300.00	0.00	33.33	-66.67	-75.00
Player 10	PLA	2	2	3	2	0.00	50.00	0.00	50.00	0.00	-33.33
Player 11	PLA	1	4	2	1	300.00	100.00	0.00	-50.00	-75.00	-50.00
Player 12	PLA	1	2	3	3	100.00	200.00	200.00	50.00	50.00	0.00
Player 13	PLA	0	6	4	3	-	-	-	-33.33	-50.00	-25.00
Player 15	PLA	0	5	4	2	-	-	-	-20.00	-60.00	-50.00
Player 16	PLA	1	6	4	3	500.00	300.00	200.00	-33.33	-50.00	-25.00
Player 17	PLA	2	4	3	2	100.00	50.00	0.00	-25.00	-50.00	-33.33
Player 18	PLA	1	3	4	2	200.00	300.00	100.00	33.33	-33.33	-50.00
Player 1	CON	1	2	3	0	100.00	200.00	-100.00	50.00	-100.00	-100.00
Player 2	CON	4	6	6	5	50.00	50.00	25.00	0.00	-16.67	-16.67
Player 3	CON	2	4	6	3	100.00	200.00	50.00	50.00	-25.00	-50.00
Player 4	CON	1	3	2	2	200.00	100.00	100.00	-33.33	-33.33	0.00
Player 5	CON	1	3	4	2	200.00	300.00	100.00	33.33	-33.33	-50.00
Player 6	CON	0	2	5	0	-	-	-	150.00	-100.00	-100.00
Player 8	CON	3	5	4	3	66.67	33.33	0.00	-20.00	-40.00	-25.00
Player 9	CON	0	3	5	0	-	-	-	66.67	-100.00	-100.00
Player 10	CON	2	2	3	1	0.00	50.00	-50.00	50.00	-50.00	-66.67
Player 11	CON	2	5	5	2	150.00	150.00	0.00	0.00	-60.00	-60.00
Player 12	CON	2	5	6	3	150.00	200.00	50.00	20.00	-40.00	-50.00
Player 13	CON	2	4	4	3	100.00	100.00	50.00	0.00	-25.00	-25.00
Player 15	CON	1	3	2	2	200.00	100.00	100.00	-33.33	-33.33	0.00
Player 16	CON	3	4	5	4	33.33	66.67	33.33	25.00	0.00	-20.00
Player 17	CON	3	5	4	3	66.67	33.33	0.00	-20.00	-40.00	-25.00
Player 18	CON	1	2	5	1	100.00	400.00	0.00	150.00	-50.00	-80.00
